# Grading images for diabetic retinopathy: tips and guidelines

**Published:** 2023-07-07

**Authors:** Catherine Jamison, Laura Cushley, Katie Curran, Tunde Peto

**Affiliations:** 1 Senior Ophthalmic Image Analyst: Centre for Public Health, Queen's University Belfast, Belfast, UK.; 2 Research Assistant: Centre for Public Health, Queen's University Belfast, Belfast, UK.; 3 Research Fellow: Centre for Public Health, Queen's University Belfast, Belfast, UK.; 4 Professor of Clinical Ophthalmology: Centre for Public Health, Queen's University Belfast, Belfast, UK.

**Diabetic retinopathy screening relies on the accurate grading of retinal images so that patients can receive the care they need. Here is how.**

This article was written as a short guide to grading retinal images for diabetic retinopathy (DR). It is mainly for people starting their grading journey, but it will also be helpful for experienced graders who want to improve upon their current techniques.

## Tips for learning and quality control

DR screening relies on graders accurately identifying patients with DR, so graders must be trained according to the relevant guidelines and chosen grading classification. There must also be a system in place for quality assurance to ensure that the accuracy of grading is maintained.

### The ‘think out loud’ approach

The ‘think out loud’ or ‘think aloud’ approach can be useful for training. When using this approach, both the expert grader and the trainee graders say out loud what they are observing and thinking when examining a fundus image for DR. When an **expert grader** does this in the presence of trainee graders, they are modelling the grading process and supporting the trainees to develop their own critical thinking skills. The expert grader can also stop and respond to questions.

When the **trainee graders** say out loud what they are seeing and thinking, this helps the expert grader to better understand their thinking processes and identify where improvements are needed. For example, a trainee grader may mistake exudates for drusen. If this happens, the expert grader can acknowledge the error and explain the differences between the two signs.

Remember: it is very important to work together within your team and learn from each other. Enjoy any teaching you can and remember there are always people who can help. Get experience – the more you grade, the better you will get, and your confidence will grow. So keep grading!

### Quality assurance

Different levels of quality assurance can be used, depending on how well established a DR screening programme is. Smaller, opportunistic DR screening programmes could introduce internal checks; for example, a proportion of the images can be double checked by a second grader to ensure agreement.

More formal systems can be introduced as programmes develop and become more systematic. An example of this is the International Test and Training (iTAT) system (**bit.ly/iTAT-DR**). Registered graders receive a monthly set of images to grade and are given automated feedback on their performance.

The Diabetic Retinopathy Network (DR-NET) offers free access to the iTAT system to graders working in any of its 32 member programmes. Graders also have access to DR-NET online workshops and quarterly DR-NET ‘Grading Grand Rounds’, during which retinal images are shown and graded anonymously by the participating graders. The ‘Grading Grand Rounds’ also allows graders to meet and discuss pertinent issues. DR programmes in low- and middle-income countries can request to become members of the DR-NET, free of charge, by registering on **www.dr-network.org**.

## Tips for grading retinal images

### Tip 1. Have a system

To be thorough and efficient as a grader, it is best to have a system when analysing a retinal image. Your system does not need to be the same as that of your fellow grader, provided that you examine all the key areas of the retina.

Using the same method every time makes it much less likely that you will forget to look at any one area.

**Figure 1:**
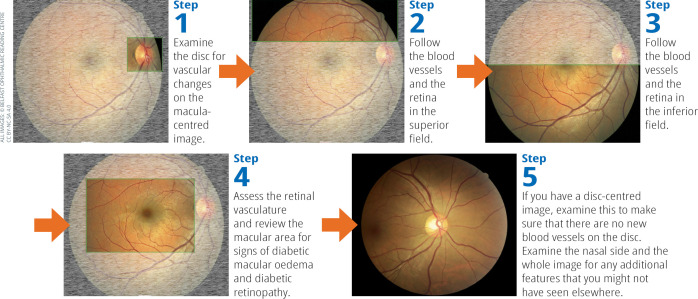
An example of a systematic approach to grading.

We use the method shown in Figure 1:

### Tip 2. Use the red-free option

**Figure 2:**
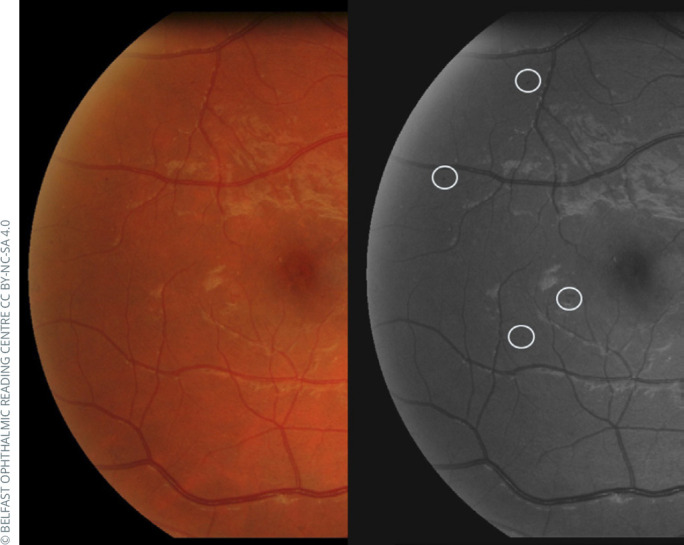
When viewing the image in black and white, microaneurysms can be seen.

Red-free is a vital and helpful tool. Even as an experienced grader, an image may look like there is no pathology at all, but when red-free is applied a microaneurysm suddenly becomes apparent (Figure 2). Even when using ordinary image-viewing software to grade an image, it is usually possible to view an image red-free by turning the colour on the image down to 0 or by applying a black-and-white filter. If possible, red-free should always be used to double-check an image.

You can zoom in and magnify the image, but make sure that the features are still visible, and the image is not too pixelated. It is best practice to zoom out again and double-check that what you have seen on the magnified image is still visible on the normal-size image.

### Tip 3. Look beyond the disc area

**Figure 3:**
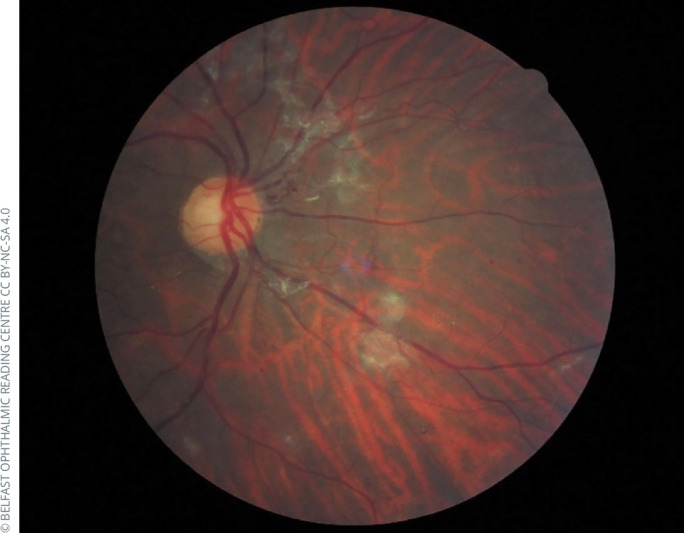
New vessels can be seen beyond the disc area.

If you are using both macula-centred and disc-centred images, always examine the area nasal to, or beyond, the optic disc. This is an area where new vessels are often observed (Figure 3). Pathology can sometimes be seen in this area, even when the entire macula-centred image appears to be pathology free.

### Tip 4. Evaluate the overall level of retinopathy

Look at the overall level of retinopathy. If an eye gives you the impression of a moderate to severe level of DR, look carefully for new vessels or other proliferative features. Always evaluate the vasculature carefully. Note that there can be new vessels even when there are no other features of DR.

### Tip 5. Know the difference between intraretinal microvascular abnormalities (IRMA) and new vessels

**Figure 4:**
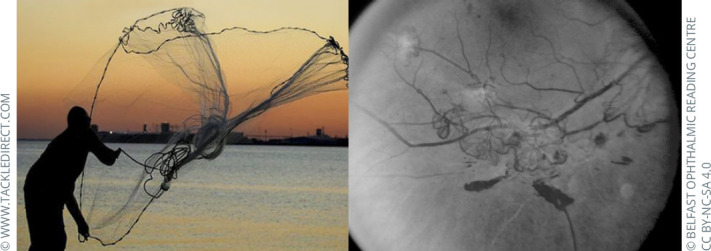
New vessels look like the casting-out of a fishing net.

The most difficult decision for any grader, no matter how experienced, is the decision between IRMA and new vessels. Some hints include:

- New vessels might look like a traditional fishing net being cast out (Figure 4), whereas IRMA tend to be more angular and irregular in appearance.
- If they are on the disc, they are new vessels
- If they cross over the top of the retinal vessels, then they are new vessels

## Other pathologies that mimic DR

It is worth taking some time to study images of other pathologies which can be similar in appearance to some DR features. This avoids grading DR when it is not the case. Features which commonly are misinterpreted include:

- Drusen
- Fibrosis and myelinated nerve fibres
- Atrophic patches and laser scars
- Reflex
- Artefacts

### Drusen

Drusen are extracellular deposits found at the retinal pigment epithelium layer. Smaller drusen can be mistaken for hard exudates and larger drusen can be mistaken for cotton wool spots. See Table 1 for comparisons between the three signs.

**Table 1:** Differences between hard exudates, drusen and cotton wool spots.

	Hard exudates	Drusen	Cotton wool spots
	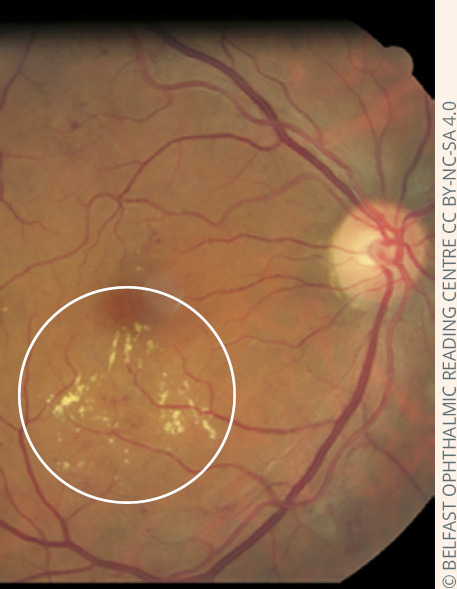	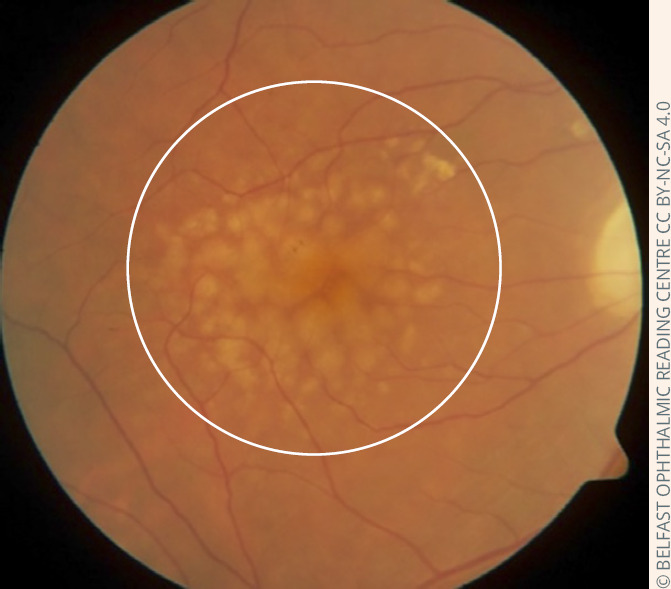	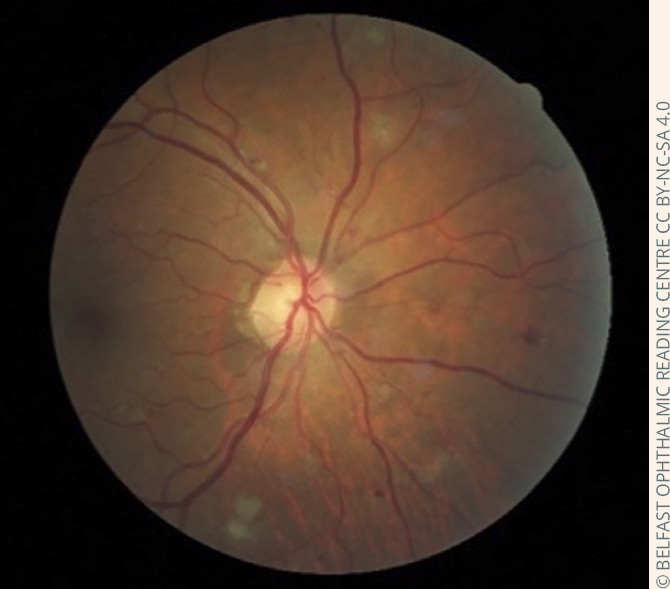
**Sign of DR?**	Yes	No	Yes in the context of having other DR features as well
**Shape and size**	Irregular	Symmetrical	Larger than most drusen
**Brightness & colour**	Bright and clear	Dull, milky-white	Milky-white
**Location**	Anywhere in the retina	Predominately in the central macula, but may also be found in the posterior pole of the fundus	Anywhere in the retina
**Depth**	At the level of the vasculature	At the level of the retinal pigment epithelium (RPE) either exterior (towards the choroid) or interior (towards the retina) of the RPE	Nerve fibre layer of the retina

### Fibrosis and myelinated nerve fibres

**Figure 5:**
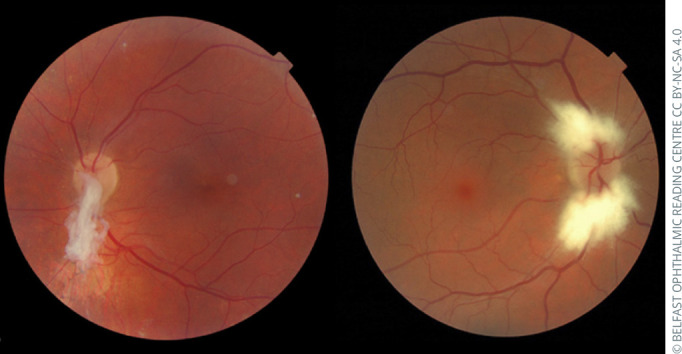
An example of fibrosis (left) and myelinated nerve fibres (right).

Fibrosis and myelinated nerve fibres tend to be found radiating out from the disc (Figure 5), although they can be found anywhere on the fundus. Fibrosis can have a whitish appearance, anywhere in the fundus, whereas myelinated nerve fibres are linear, streaky and follow along the lines of the nerve fibres. Myelinated nerve fibres are not a feature of DR, whereas fibrosis is a feature of proliferative retinopathy.

### Atrophic patches and laser scars

**Figure 6:**
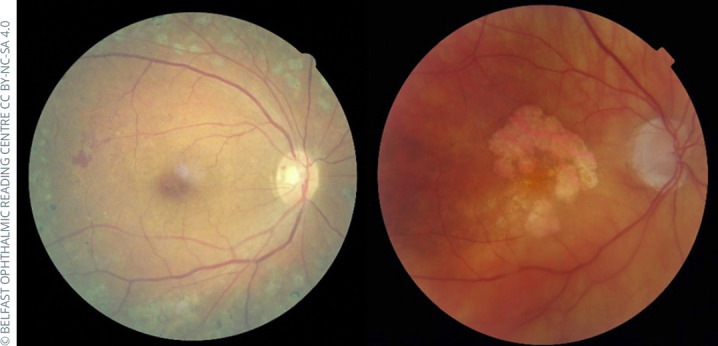
Examples of laser scars (left) and atrophy (right).

Small patches of atrophy and laser scars (Figure 6) are areas where the outer layer of the retina has been destroyed either by laser treatment creating a scar, or by a disease that led to retinal death. Laser scars are likely to be smaller than atrophy patches and may be in an unnatural, grid-like pattern in the macula or in areas of the periphery. The appearance of the laser scar depends on the laser used and the time elapsed since the laser treatment was carried out. Laser scars are more likely to be peripheral and are unlikely to be very close to the fovea.

### Reflex

**Figure 7:**
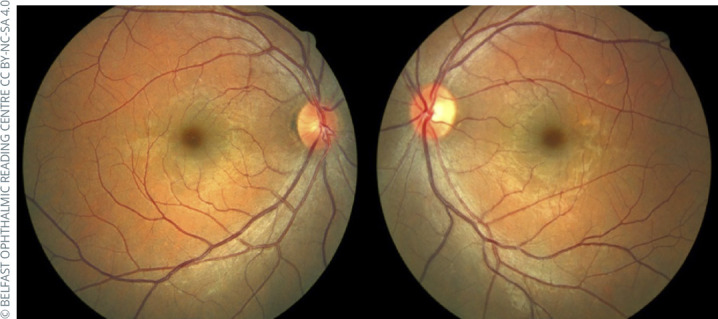
Examples of reflex.

Younger people will have a shiny reflex visible on the retina (Figure 7). This has a wispy, smoke-like appearance or sheen and is not pathology of any kind. Sometimes these reflexes can be mistaken for exudates.

### Artefacts

**Figure 8:**
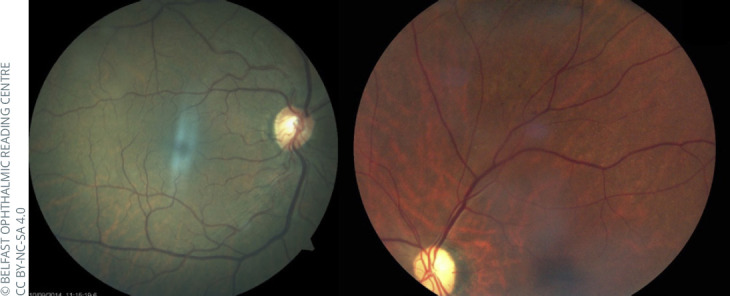
Examples of artefacts on a retinal image.

Be aware of artefacts and how to spot them. Artefacts are caused by any object on the lens that may obscure the image (Figure 8, left), such as eyelashes, dust, saliva droplets from speaking over the lens, or fingerprints. Dirt on the camera appears as circular, greyish marks (Figure 8, right).

If you are unsure whether something is an artefact, look at the disc-centred image or images of the other eye. If the marks appear in the same location on any other image, then they are artefacts. However, if the marks move with the eye and appear on the same retinal location, then they are likely to be real changes.

